# Serbian Language version of the Modified Checklist for Autism in Toddlers, Revised, with Follow-Up: Cross-Cultural Adaptation and Assessment of Reliability

**DOI:** 10.1038/srep38222

**Published:** 2016-12-01

**Authors:** Mia Carakovac, Jelena Jovanovic, Marko Kalanj, Nenad Rudic, Olivera Aleksic–Hil, Branko Aleksic, Itzel Bustos Villalobos, Hideki Kasuya, Norio Ozaki, Dusica Lecic–Tosevski, Milica Pejovic–Milovancevic

**Affiliations:** 1 Faculty of special education and rehabilitation, University of Belgrade, Visokog Stevana 2, 11000 Belgrade, Serbia; 2 Institute of Mental Health, Palmoticeva 37, 11000 Belgrade, Serbia; 3 Department of Psychiatry, Nagoya University Graduate School of Medicine, 65 Tsurumai-cho, Showa-ku, Nagoya, Aichi-ken 466-8550, Japan; 4 Office on international affairs, Nagoya University Graduate School of Medicine, 65 Tsurumai-cho, Showa-ku, Nagoya, Aichi-ken 466-8550, Japan; 5 School of Medicine, University of Belgrade, Dr Subotica 2, 11000 Belgrade, Serbia; 6 Serbian Academy of Sciences and Arts, Knez Mihailova 35, 11000 Belgrade, Serbia.

## Abstract

Early detection of Autism Spectrum Disorder (ASD) has proven to be of high significance, however there is a limited availability of ASD screening tools in Serbian language. In this study we aim to translate, assess reliability and, in part, test the applicability of Modified Checklist for Autism in Toddlers, Revised, with Follow-Up (M-CHAT R/F) in Serbian Healthcare environment. We screened 128 children in three primary healthcare centres and 20 children in a tertiary psychiatric center, using M-CHAT R/F translated into Serbian language, between December 2014 and October 2015. At the end of the screening process 80% of participants in the risk group screened positive for ASD, while in the control group 4 (3.1%) participants screened positive, with a mean total scores of 8.25 and 0.66 respectively. The Cronbach’s α coefficient was 0.91 and Guttman’s λ6 was 0.93. Test – retest reliability was deemed as acceptable, and no significant correlation was found between M-CHAT-R/F scores and Epworth Sleepiness Scale for children scores. The Serbian version of the M-CHAT-R/F has shown satisfactory reliability. We can therefore assert that it is a reliable tool for identifying ASD and it can be used in clinical practice to improve early detection, assessment and treatment.

Autism spectrum disorder (ASD) is a common neurodevelopmental disorder manifested by impairments in social interaction and communication, as well as the presence of repetitive and restricted behaviours/interests^1^. In 2010, the prevalence of ASD in the United States was estimated at 14.7 cases per 1000 children, or 1 in 68 children, with substantial variability in estimates by region, sex, and race/ethnicity^2^. Diagnosis of ASD relies on set of subjective qualitative or semi-qualitative criteria, however an objective clinical test that can facilitate diagnostic process is not available^3^. Furthermore, it is well established that early intervention is associated with better clinical outcomes in persons with ASD^4^, raising the hope that treatment during the initial phase of illness could prevent the development of more severe form of disorder and thus reduce risk of chronic symptoms and disability. Therefore, for ASD in particular, it is crucial to improve paediatrician skills in early screening of possible developmental delay, more specifically ASD^5^. The American Academy of Paediatrics (AAP) recommends that ASD-specific standardize screening should be performed at 18- and 24-month preventive check-ups^6^; even though a 2009 survey among paediatricians found that only 28% routinely used it and most common barriers included lack of time and lack of familiarity with screening tools^7^.

Although autism is hard to diagnose before 36 months, symptoms often surface between 12 and 18 months^8^, there is a large delay between the first parental concerns and the diagnosis^9^. Recognition and diagnosis of ASD also depends on the parents’ education level and attitude^10^. While the average reported age of ASD diagnosis is 3.9 to 5.7 years depending on research design^11^,^12^,^13^, many parents of diagnosed ASD children recall concerns in the years prior to ASD initial diagnosis, and as many as 50% of them suspected their children had problems even by the end of their first year. Not to mention, that children with milder symptoms are often diagnosed much later.

Early detection of ASD proved to be of high significance, since early implementation of intensive treatment strategies leads to a better prognosis for the affected child. Moreover, early detection helps with reducing family stress and societal burden^9^. Due to lack of reproducible biological markers for determination of an ASD diagnosis, early detection has been based on a combination of different strategies. Some of those strategies include developmental surveillance through expert casual assessment of communication, social and play skills, and consideration of parental concerns^13^,^14^. Paediatricians need support as well, in the form of sensitive screening tools, in order to provide better care, and if needed, refer the child to other specialists^6^.

ASD-specific screening tools have been classified into level 1 and level 2 screening tools. Level 1 screening tools have been designed for primary health care setting^6^, and level 1 screening applies to all children regardless of risk status (i.e., “universal” screening). In contrast, level 2 screening is targeted at children already identified as being at increased risk (e.g., due to a positive family history, concerns raised by parents or clinicians, identification by a level 1 screener).

One of the first available level 1 screening tools for ASD was the Checklist for Autism in Toddlers (CHAT), developed by Baron-Cohen *et al*. in 1992. As a more sensitive alternative to this questionnaire, Robins *et al*. constructed in 2001, for the needs of the American healthcare system, a Modified Checklist for Autism in Toddlers (M-CHAT)^15^. This adaptation eliminated the physician observation section and expanded on the parent report items. Extensive validation of this instrument pointed towards the need for a follow-up, done by phone or other methods, of children with positive screening, in order to minimize the number of false positive cases. As to avoid the false negative cases, the minimum score for a positive screening result was significantly lowered. In the meantime, the authors performed a revision of the questionnaire, decreasing the number and rephrasing certain items. Modified Checklist for Autism in Toddlers, Revised with Follow-up (M-CHAT-R/F) was designed by Robins *et al*. in 2009^16^,^17^ and it has since been translated into 35 languages or dialects.

M-CHAT-R/F is a two-stage ASD screening instrument intended for parents of 16 to 30 months old children. It contains 20 items for which the parent choses a YES/NO answer. In case the screening proves positive (3 or more positive answers), a structured Follow-Up interview (FUI) is conducted with the parent, in order to attain additional information regarding examples of risk behaviour of the child and to determine the need for further diagnostics, monitoring and evaluation. For children whose scores are 8 and above, it is acceptable to bypass the Follow-Up and refer them immediately for diagnostic evaluation and eligibility evaluation for early intervention^16^.

Primary health care institutions in Serbia are organized in each municipality; each child is assigned a paediatrician as a primary health care chosen doctor for the entire school age, and attends regular preventive health screening visits during early development. Paediatricians, if necessary, can refer children to secondary or tertiary health care levels, depending on the problem. Developmental problems in children are fully assessed mostly in tertiary health care institutions, most of which are specifically oriented to a particular area. In view of limited availability of level 1 screening tools in Serbian language, as well as sparse data of their application in Serbian primary health care setting, we sought to translate, assess reliability and, in part, test the applicability of M-CHAT-R/F as an ASD screening tool in Serbian clinical setting.

## Methods

### Preparation of Serbian M-CHAT/R-F version

After obtaining permission from the authors, the M-CHAT-R/F was translated from English to Serbian, independently by two bilingual researchers, and the translations were compared and combined. A back translation was also performed by a third party, with the result comparable to the original. Slight adaptation of wording was required due to languages differences. The research was conducted in three primary health care centres, and in one tertiary psychiatric centre with a child and adolescent psychiatric unit within it, between December 2014 and October 2015.

For the purpose of the study primary heath care centres were randomly selected. The tertiary psychiatric centre was chosen because it is one of the institutions where children with suspected developmental problems are referred to for assessment and treatment, within the child and adolescent psychiatry unit. Organization of Serbian health care system is at present time constructed in such a way that this level of specialized health care service was deemed most adequate in providing us with the easiest access to children, within the Serbian health care system, in risk of developmental delays or disorders. The ethics committees of Institute of Mental Health in Belgrade and School of Medicine, University of Belgrade approved the experimental protocols used in the current study. All experiments were performed in accordance with the committees’ guidelines and regulations. Written informed consent was obtained from all participants or legal guardians. Each patient’s capacity to provide consent was confirmed by a family member or legal guardians when needed. Individuals with a legal measure of reduced capacity were excluded.

### Participants

The control group was comprised of 128 participants in primary healthcare setting, some of which were children attending a scheduled preventive health-screening visits at 18 and 24 months of age, and some were children captured during a spontaneous paediatrician visit, between 16 and 30 months of age.

The risk group included 20 children, referred for evaluation and diagnostics to a specialized tertiary psychiatric institution with child and adolescents psychiatry service, with suspicion of developmental delay or disorder, including ASD.

### Procedure

A questionnaire package was compiled, containing a written consent form, a general questionnaire covering child demographics, ASD risk factors and previous development, a Serbian version of M-CHAT-R/F, as well as Epworth Sleepiness Scale for children^18^, to assess the discriminant validity. Trained health care professionals administered the questionnaire packages.

The procedure included two stages. During stage one, after being provided with basic information on the research, the parents were asked to sign a consent form and were left to fill out the questionnaires. This usually took 20 to 30 minutes, including the 5 to 10 minutes required to complete the M-CHAT-R/F. Scoring of the M-CHAT-R/F was conducted on the spot.

The stage two followed in instances when the M-CHAT-R/F score was between 3 and 7. In these cases the Follow-up interview (FUI) was implemented, which usually took under 10 minutes. If score upon Follow-Up was 0–1, child has screened negative. If M-CHAT-R/F score remained at 2 or higher, the child has screened positive, and the attending paediatrician or child psychiatrist was notified. A number of randomly chosen participants (N = 26) were contacted via telephone several weeks later and asked to complete again the first stage of M-CHAT-R/F, in order to evaluate test-retest reliability.

## Results

A summary of participants’ demographic characteristics is given in Table 1.

No significant differences between groups were observed in regards to sex, age, number of siblings and parents’ age at childbirth. Mean gestational age at birth was found to be lower in the risk group (F(1,136) = 9.63, p = 0.002), indicating a possible risk factor for onset of neurodevelopmental disturbances.

### Results of screening

At stage one, in the risk group, 15 (75%) of participants were classified as high risk, 1 (5%) as medium risk and 4 (20%) as low risk for ASD, while in the control group, 1 (0.8%) participant was classified as high risk, 9 (7%) as medium risk and 118 (92.2%) as low risk. Mean total score at stage one was 8.45 (σ = 4.92) in the risk group, and 0.79 (σ = 1.49) in the control group. After the follow-up interview, in stage two, 16 (80%) participants in the risk group screened positive for ASD, with a mean total score of 8.25 (σ = 4.88), while in the control group 4 (3.1%) participants screened positive, with a mean total score of 0.66 (σ = 1.37).

The frequencies of participants in both groups who scored a “fail” on each item are given in Table 2.

Participants in the risk group failed almost all items with greater frequency than participants in the control group. In both groups, relatively low frequencies of “fail” responses have been observed on motor items (items 4, and 20), not surprising, considering these items were created as foils^17^ and do not assess ASD. A markedly high number of participants from the risk group failed items whose content relates to socialization (items 7, 15, and 16).

### Psychometric properties

M-CHAT-R/F displayed excellent internal consistency across all items, with Cronbach’s α = 0.91 and Guttman’s λ6 = 0.93 (λ6 being based on the amount of variance in each item that can be accounted for the linear regression of all of the other items). Given the heterogenous content of the items, Cronbach’s α is suprisingly high; however, corrected item – total correlations for motor items were in the order of 0.2, or non-significant, while corrected item – total correlations for all other items were in the range of 0.3–0.8, averaging at 0.67, accounting for high internal consistency.Kaiser-Meyer-Olkin (KMO) measure of sampling adequacy was 0.89, which can be considered as satisfactory, given the heterogeneous content of the items. Correlation between total first-stage scores at the initial examination and total scores 4 weeks later was r = 0.80, p < 0.001, indicating acceptable test – retest reliability. No significant correlation was found between M-CHAT-R/F scores and Epworth Sleepiness Scale for children scores.

## Discussion

This is the first study to assess reliability M-CHAT/R-F in Serbia and our paper outlines the two phases of this process: translation and reliability analysis. Our study suggests that M-CHAT/R-F is sufficiently reliable. Using a four-week test-retest method, total scores of the Serbian version of M-CHAT/R-F at two time points were significantly correlated; measures of internal consistency were also satisfactory. Furthermore, using Epworth Sleepiness Scale for children we have shown that Serbian version of M-CHAT/R-F has divergent validity.

Several studies have used the M-CHAT in the general population as a level 1 screening instrument during well-child visits, but using the Follow-up interview reduced the screen positive rate by 65–80%^19^.

Some clinicians believed that M-CHAT (without FUI) is over inclusive and the later prevalence of ASD estimated around 0.9% lower than the M-CHAT screen-positive rate (6.4%)^20^. This was a particular reason why we selected version with FUI and translated it into Serbian. This specific interview is a screening instrument for the general population and it is up to the investigator, in this case predominately pediatrician, to weight the balance between the false negative and false positive results.

We believe that screening procedure with M-CHAT/R-F should be used in routine practice, as already is a standard procedure in Spain^9^. In USA, this instrument has demonstrated to be an effective tool for screening of low-risk toddlers and American Academy of Child and Adolescent Psychiatry (AACAP) supports the screening of ASD in young children and, in some instances, in older ones, as well^21^. Children should be rescreened if they are younger then 24 months, as recommended by American Academy of Pediatrics, and authors of the instrument suggested that all children who continue to show risk after Follow-Up should be screened, and that this is anticipated to be approximately one third of all of the children who complete the Follow-Up based on initial risk, and will need referral for further diagnostic procedure and early interventions^16^. However, screening information should be carefully communicated to the parents and in addition to education on how to use the instrument, the pediatrician should acquire knowledge about motivational strategies for parents/caregivers to follow up diagnostic and treatment procedures.

M-CHAT/R-F administration should start with the pediatrician and/or member of developmental team within the primary health care center. They have to introduce the characteristics of this screening instrument to parents. We clearly see the need for coordination between the health services and specific early intervention, either with health care, education or social welfare system.

Implementing standardized screening procedure can greatly decrease the time in which the children and their parents are referred to the centers where they can get the proper diagnostic and suggested treatment. Children who receive early interventions at younger ages have improved outcomes in different developmental domains (communication, language, and social skills)^19^. Also, in the creation of policies devoted to early development it is important to have standardized screening tools in order to be able to identify children at risk and to further promote developing of centers for early intervention.

## Limitations

Primary limitations of our study are relatively small sample sizes, and the fact that the risk group was selected solely on basis of referral for diagnostic evaluation, with suspicion of ASD or developmental delay, and not on basis of a confirmed ASD diagnosis, which would have enabled calculating instrument sensitivity and specificity on our sample. However our design corresponds fully to the fact that we conducted a naturalistic study, in a ‘real-life’ clinical setting. To overcome these limitations follow-up research with more sophisticated design will be required, taking into consideration opinions and comments of mental health professionals who administrated the Serbian translation of M-CHAT-R/F. Finally, in the current study we did not include a group diagnosed with ASD in order to have a comparison group.

## Conclusion

The Serbian version of the M-CHAT-R/F has shown satisfactory reliability. We can therefore assert that it is a reliable tool for identifying ASD and it can be used by healthcare professionals in their clinical practice to improve early detection, assessment and treatment for children with high scores. Early detection of ASD will result in early identification and consequently early intervention. Many studies already have shown that early interventions are associated with improved social behavior, language improvement and decrease of other ASD symptoms.

## Additional Information

**How to cite this article**: Carakovac, M. *et al*. Serbian Language version of the Modified Checklist for Autism in Toddlers, Revised, with Follow-Up: Cross-Cultural Adaptation and Assessment of Reliability. *Sci. Rep.* **6**, 38222; doi: 10.1038/srep38222 (2016).

**Publisher’s note:** Springer Nature remains neutral with regard to jurisdictional claims in published maps and institutional affiliations.

## Figures and Tables

**Table 1:** Sample characteristics.

	Group	
	Control (N = 128)	Risk (N = 20)
Sex ratio (male %: female %)	57:43	70:30
Mean age in months (standard deviation)	22.25 (3.94)	23.53 (4.26)
Mean mother’s age at childbirth (standard deviation)	30.4 (5.29)	31.2 (3.65)
Mean father’s age at childbirth (standard deviation)	33.92 (6.8)	34.47 (4.64)
Mean gestational age (weeks) at birth (standard deviation)	38.57 (2.06)	36.82 (2.87)
Number of siblings (mode)	1	1

**Table 2:** Frequencies of “fail” scores per item in control and risk group.

Items	Group	
	Control	Risk
1.	3 (2.3%)	12 (60%)
2.	5 (3.9%)	7 (35%)
3.	5 (3.9%)	9 (45%)
4.	1 (0.8%)	1 (5%)
5.	11(8.6%)	5 (25%)
6.	6 (4.7%)	10 (50%)
7.	1 (3.1%)	16 (80%)
8.	3 (2.4%)	7 (35%)
9.	2 (1.6%)	10 (50%)
10.	2 (1.6%)	10 (50%)
11.	2 (1.6%)	10 (50%)
12.	1 (0.8%)	4 (20%)
13.	10 (7.8%)	6 (30%)
14.	1 (0.8%)	8 (40%)
15.	2 (1.6%)	14 (70%)
16.	9 (7%)	16 (80%)
17.	4 (3.1%)	9 (45%)
18.	3 (2.3%)	12 (60%)
19.	10 (7.8%)	9 (45%)
20.	1 (0.8%)	3 (15%)

Description of items avalable online (http://mchatscreen.com).
